# Dataset on gene expressions affected by simultaneous knockdown of Hedgehog and Dpp signaling components in embryos of the spider *Parasteatoda tepidariorum*

**DOI:** 10.1016/j.dib.2019.105088

**Published:** 2020-01-03

**Authors:** Hiroki Oda, Yasuko Akiyama-Oda

**Affiliations:** a Laboratory of Evolutionary Cell and Developmental Biology, JT Biohistory Research Hall, 1-1 Murasaki-cho, Takatsuki, Osaka, 569-1125, Japan; b Laboratory of Biohistory, Department of Biological Sciences, Graduate School of Science, Osaka University, Japan; c Microbiology and Infection Control, Osaka Medical College, Takatsuki, Osaka, Japan

**Keywords:** Axis formation, Pattern formation, Embryology, Arthropod, Emerging model organism, RNA interference, Microarray, Signaling pathway

## Abstract

Simultaneous, parental RNA interference (pRNAi) mediated knockdown of Hedgehog and Decapentaplegic (Dpp) signaling components, *Pt-patched* (*Pt-ptc*) and *Pt-dpp*, respectively, exhibited serious defects in the formation of the major embryonic axes in the model spider *Parasteatoda tepidariorum*. This paper describes a dataset of a custom oligonucleotide two-color microarray analysis that was carried out to compare the transcript expression levels between untreated (normal) and *Pt-ptc* + *Pt-dpp* double pRNAi embryos at late stage 5. Array spots that showed the intensity ratio of [*Pt-ptc* + *Pt-dpp* double pRNAi]/[normal] <0.6 were categorized as positive. The expressions of most, not all, of the transcripts related to the positive array spots were examined in embryos by whole-mount *in situ* hybridization. Some of the stained embryos showed distinct patterns of gene expression. The data presented may be useful for characterizing the mechanisms of embryonic patterning in spider embryos.

**Table:** Specifications Table

Subject	Developmental Biology
Specific subject area	Axis formation in animal embryos
Type of data	Table Image
How data were acquired	Custom oligonucleotide, two-color microarray; whole-mount *in situ* hybridization
Data format	Raw Analyzed
Parameters for data collection	No biological or technical replicates, with positive and negative controls
Description of data collection	Total RNAs that were extracted from late stage 5 embryos produced by the same parents before and after, respectively, *Pt-ptc* + *Pt-dpp* double pRNAi treatment were used for microarray analysis.
Data source location	Osaka, Japan
Data accessibility	For the microarray data, Repository name: Gene Expression Omnibus (GEO) at NCBI Data identification number: GSE112435 Direct URL to data: https://www.ncbi.nlm.nih.gov/geo/query/acc.cgi?acc= GSE112435 For the WISH images, Repository name: Mendeley Data Data identification number: c7cfhyd2p3 Direct URL to data: https://data.mendeley.com/datasets/c7cfhyd2p3/3

**Table:** 

**Value of the Data** • These data are useful for identifying the candidate genes whose expression is regulated by Hh and/or Dpp signaling in *P. tepidariorum* embryos. • These data are informative for researchers who are interested in mechanisms of axis formation in animal embryos and/or those of pattern formation mediated by cell signaling pathways. • These data can be used for discovering novel regulatory networks of genes involved in embryonic patterning.

## 1 Data

**Fig. 1:**
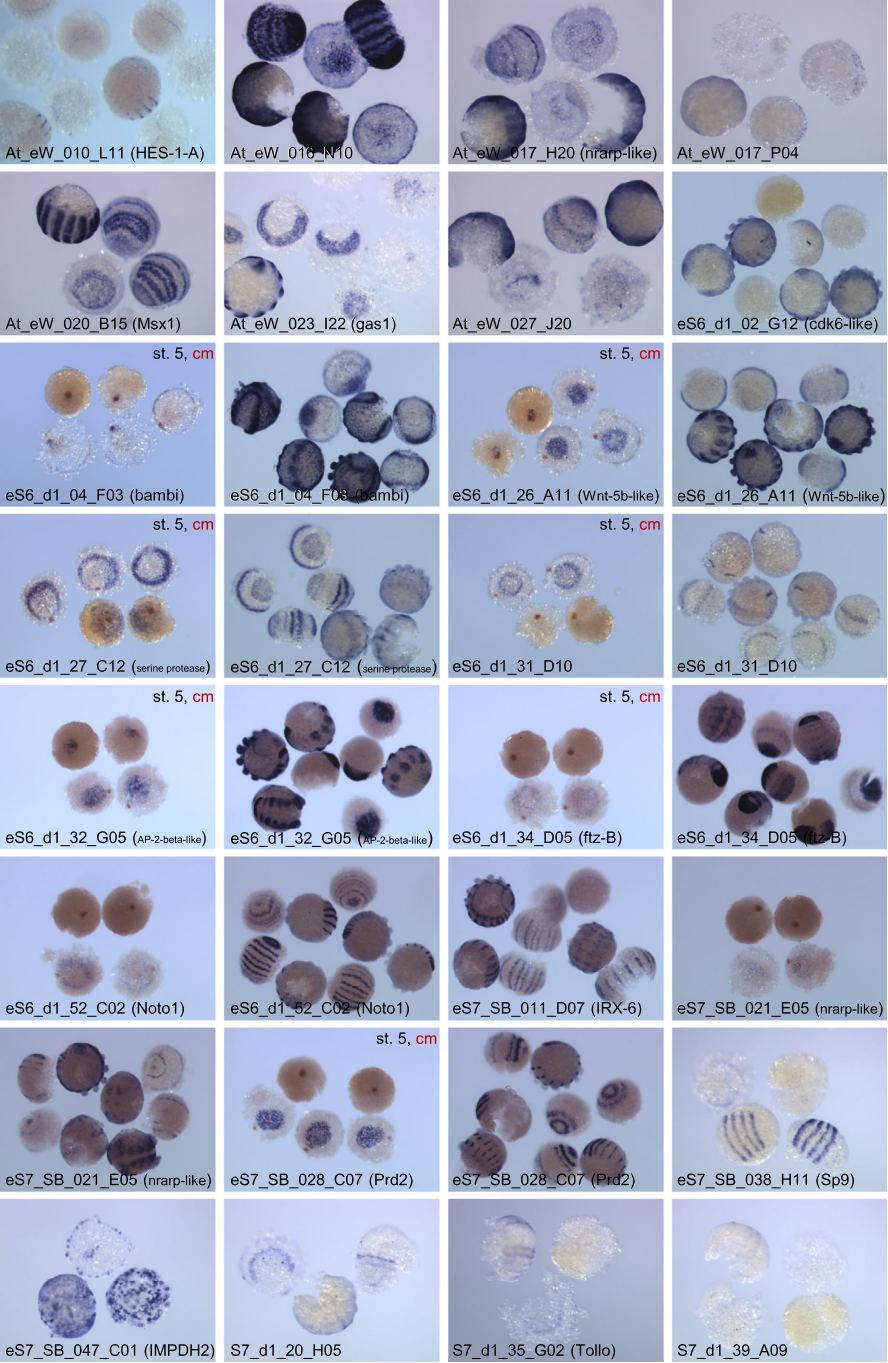
**Staining of stage 5−8 embryos for selected transcripts by WISH.** The identity of EST clones that were used for the synthesis of RNA probes is indicated in each panel. Some panels show stage 5 embryos additionally stained in red for a cumulus cell marker (cm).

We obtained embryos that showed serious defects in axis formation and extra-embryonic differentiation caused by simultaneous, parental RNA interference (pRNAi) mediated knockdown of *Pt-patched* (*Pt-ptc*) and *Pt-decapentaplegic* (*Pt-dpp*) (Movie S1), as was predictable from results of our previous experiments [1,2]. In *Pt-ptc* + *Pt-dpp* double pRNAi embryos, the migration of cumulus mesenchymal cells was impaired as observed in *Pt-ptc* single pRNAi embryos but no ectopic extra-embryonic differentiation occurred unlike in the *Pt-ptc* single pRNAi embryos [2]. This was presumably due to the simultaneous knockdown of *Pt-dpp*, which has been shown to be involved in the induction of extra-embryonic differentiation [1]. Using Combimatrix custom microarrays previously described [3], we compared the levels of the transcript expressions between untreated (normal) and *Pt-ptc* + *Pt-dpp* double pRNAi embryos at late stage 5. The microarray dataset deposited in the GEO Database at NCBI (GSE112435) consists of a data table showing the details of probe sequences for array spots (Platform: GPL11390 and GPL11391) and one showing the normalized signal intensity ratio of [*Pt-ptc* + *Pt-dpp* double pRNAi]/[normal] for each array spot (Sample: GSM3070092 and GSM3070093). Values of the [*Pt-ptc* + *Pt-dpp* double pRNAi]/[normal] intensity ratio from control probes are shown in Table 1. Array spots that showed the intensity ratio of [*Pt-ptc* + *Pt-dpp* double pRNAi]/[normal] < 0.6 were categorized as positive, and are listed in Table 2. Additional information about the control and positive array spots, including probe sequences, gene models, gene accessions, and notes based on the previously described developmental transcriptomes [4], is available in Supplementary Tables 1 and 2 (Tables S1 and S2), respectively. The expressions of most, not all, of the transcripts related to the positive array spots were examined in embryos by whole-mount *in situ* hybridization (Table S2). Some of the stained embryos showed distinct patterns of gene expression, which were photographed and are displayed in Fig. 1. The original images are available in the Mendeley data repository [5] and in the searchable databases of the Biohistory Research Hall (BRH) Data Resources (https://www.brh2.jp).

**Table 1:** Values of the [*Pt-ptc, Pt-dpp* RNAi]/[normal] intensity ratios from control probes in the microarray analysis.

Array No.	REF_ID	Ratio	EST clone ID	Sequence accession	RefSeq Gene ID	Description
1	6978	0.998	At_eW_003_D02	FY217447	LOC107439705	catenin alpha
1	7997	1.469	At_eW_003_D02	FY217447	LOC107439705	catenin alpha
1	9278	0.867	At_eW_003_D02	FY217447	LOC107439705	catenin alpha
1	11582	1.144	At_eW_003_D02	FY217447	LOC107439705	catenin alpha
2	6342	0.988	At_eW_003_D02	FY217447	LOC107439705	catenin alpha
2	11607	1.047	At_eW_003_D02	FY217447	LOC107439705	catenin alpha
1	4354	1.054	eS7_003_G08	FY376809	LOC107441347	elongation factor 1-alpha
1	10730	1.077	eS7_003_G08	FY376809	LOC107441347	elongation factor 1-alpha
2	430	1.083	eS7_003_G08	FY376809	LOC107441347	elongation factor 1-alpha
1	3623	1.006	eS7_SB_037_C01	FY380578	LOC107447866	histone H3.3
1	9723	0.927	eS7_SB_037_C01	FY380578	LOC107447866	histone H3.3
2	6417	0.928	eS7_SB_037_C01	FY380578	LOC107447866	histone H3.3

**Table 2:** List of array spots that showed the intensity ratios [*Pt-ptc* + *Pt-dpp* double RNAi]/[normal] of <0.6.

Array No.	REF_ID	Ratio	EST clone ID or gene name^a^	RefSeq Gene ID or GB_ACC	Description
1	450	0.580	At_eW_000_C16	LOC107441590	rap guanine nucleotide exchange factor 2-like
1	1005	0.567	At_eW_000_E06	LOC107449884	notch-regulated ankyrin repeat-containing protein-like
1	6423	0.549	At_eW_000_J22	LOC107449884	notch-regulated ankyrin repeat-containing protein-like
2	8616	0.516	eS7_SB_021_E05*	LOC107449884	notch-regulated ankyrin repeat-containing protein-like
2	10410	0.487	S7_d1_18_A10	LOC107449884	notch-regulated ankyrin repeat-containing protein-like
2	6880	0.521	S7_d1_18_A10	LOC107449884	notch-regulated ankyrin repeat-containing protein-like
1	6611	0.508	At_eW_000_F24	IABY01000175	18S ribosomal RNA gene
1	11812	0.462	At_eW_000_F24	IABY01000175	18S ribosomal RNA gene
1	7145	0.597	At_eW_000_M09		
1	8819	0.576	At_eW_002_L19	LOC107444999	epidermal growth factor receptor kinase substrate 8-like protein 2
1	919	0.442	At_eW_003_L15	LOC107438525	protein melted
1	9418	0.509	At_eW_003_L17		
1	661	0.554	At_eW_004_D03		
1	12282	0.541	At_eW_004_F24	LOC107438715	TBC1 domain family member 22B
1	339	0.547	At_eW_004_N23	LOC107439340	cilia- and flagella-associated protein 58
1	9968	0.592	At_eW_005_C14		
1	12481	0.536	At_eW_005_D02		
1	3856	0.582	At_eW_005_D07		
1	3745	0.583	At_eW_005_P05	LOC107451405	U2 small nuclear ribonucleoprotein A′
1	10076	0.598	At_eW_005_P06		
1	3583	0.544	At_eW_005_P09	LOC110282483	uncharacterized LOC110282483
1	2714	0.534	At_eW_007_J22		
1	2093	0.512	At_eW_007_M22	LOC107443747	protein SHQ1 homolog
1	2326	0.592	At_eW_008_I02	LOC107452247	uncharacterized LOC107452247
1	11478	0.563	At_eW_009_O12		
1	7942	0.589	At_eW_010_D11		
1	1482	0.567	At_eW_010_H20		
1	150	0.577	At_eW_010_L11	LOC107444253	transcription factor HES-1-A
1	6462	0.591	At_eW_011_C15	LOC107436693	transmembrane protein 165-like
1	9029	0.595	At_eW_011_D17		
1	11467	0.528	At_eW_012_N12	LOC107446959	uncharacterized LOC107446959
1	5534	0.572	At_eW_013_F08		
1	1284	0.463	At_eW_013_I14		
1	1922	0.570	At_eW_014_K24	LOC107448046	anaphase-promoting complex subunit 1
1	4791	0.527	At_eW_016_G24		
1	8895	0.573	At_eW_016_H18	LOC107445612	neurobeachin
1	8551	0.583	At_eW_016_K20	LOC107454643	fasciclin-2
1	9159	0.578	At_eW_016_L03		
1	10700	0.540	At_eW_016_N10*	LOC107452890	uncharacterized LOC107452890
1	9831	0.543	At_eW_017_A06		
1	6001	0.598	At_eW_017_H11		
1	12023	0.543	At_eW_017_H20*	IABY01007316	notch-regulated ankyrin repeat-containing protein-like
1	12159	0.506	At_eW_017_N01		
1	436	0.587	At_eW_017_P04*		
1	7805	0.595	At_eW_018_F16		
1	2925	0.565	At_eW_018_K04	LOC107447180	protein Wnt-5b-like
2	10650	0.561	*Pt-wnt5*	LOC107447180	protein Wnt-5b-like
1	3287	0.360	eS6_d1_26_A11*	LOC107447180	protein Wnt-5b-like
1	11579	0.410	At_eW_019_D19	LOC107438410	myosin regulatory light chain 2
1	7511	0.522	At_eW_019_H22	LOC107446659	pituitary tumor-transforming gene 1 protein-interacting protein
1	7367	0.528	At_eW_019_L05	IABY01019505	beta-1,4-galactosyltransferase 7-like
1	7525	0.597	At_eW_019_M01		
1	11212	0.454	At_eW_019_O17	LOC107440487	heat shock 70 kDa protein cognate 4
1	2858	0.578	At_eW_020_B15*	LOC107447678	homeobox protein MSH-D-like, Msx1
1	10173	0.552	At_eW_020_D06		
1	4395	0.571	At_eW_021_C05		
1	9232	0.516	At_eW_021_K24		
1	11068	0.538	At_eW_022_I21	LOC107446292	protein sel-1 homolog 1-like
1	5400	0.590	At_eW_023_A14	LOC107453070	uncharacterized LOC107453070
1	12508	0.501	At_eW_023_I22*	LOC107438015	growth arrest-specific protein 1
1	2821	0.493	At_eW_023_I22*	LOC107438015	growth arrest-specific protein 1
1	9995	0.548	At_eW_023_J04		
1	1123	0.593	At_eW_023_M02	LOC107441148	lipopolysaccharide-induced tumor necrosis factor-alpha factor homolog
1	12307	0.594	At_eW_024_C09		
1	5992	0.529	At_eW_024_H11	IABY01020283	
1	540	0.577	At_eW_024_P15	LOC107447475	ubiquitin-protein ligase E3A-like
1	8886	0.571	At_eW_025_M12		
1	3886	0.562	At_eW_026_K05	LOC107446429	uncharacterized LOC107446429
1	9733	0.458	At_eW_027_J20*	LOC107446595	uncharacterized LOC107446595
1	11788	0.573	At_eW_027_N08		
1	612	0.389	*Pt-dpp*	LOC107442925	bone morphogenetic protein 4-like
1	5343	0.546	eS6_d1_12_H07	LOC107442925	bone morphogenetic protein 4-like
1	6206	0.514	*Pt-cad*	LOC107437910	homeobox protein CDX-1-like
1	8638	0.463	*Pt-gataC*	LOC107448880	endothelial transcription factor GATA-2-like
1	9435	0.598	eS6_d1_01_A08		
1	9555	0.523	eS6_d1_01_C03		
1	12467	0.594	eS6_d1_01_D11		
1	6832	0.557	eS6_d1_02_C06	LOC107457141	protein capicua homolog
1	4902	0.566	eS6_d1_02_G12*	LOC107439895	cyclin-dependent kinase 6-like
1	12346	0.569	eS6_d1_03_B05	LOC107436245	polypeptide *N*-acetylgalactosaminyltransferase 1-like
1	9898	0.404	eS6_d1_03_D06		
1	6599	0.588	eS6_d1_03_D09		
1	9521	0.594	eS6_d1_04_F03*	LOC107443591	BMP and activin membrane-bound inhibitor homolog
1	7264	0.563	eS6_d1_05_E04		
1	2563	0.565	eS6_d1_09_B04	IABY01006050	
1	10790	0.590	eS6_d1_09_B09	LOC107454942	uncharacterized LOC107454942
1	6404	0.582	eS6_d1_12_D08		
1	1398	0.412	eS6_d1_13_E07		
1	8383	0.561	eS6_d1_14_A02		
1	2126	0.478	eS6_d1_15_H06		
1	4375	0.597	eS6_d1_21_A11		
1	11328	0.589	eS6_d1_23_G03	LOC107449017	1-acyl-sn-glycerol-3-phosphate acyltransferase beta
1	3476	0.517	eS6_d1_23_H04		
1	2074	0.575	eS6_d1_25_E09		
1	107	0.592	eS6_d1_26_H06		
1	6730	0.588	eS6_d1_27_C12*	LOC107437911	serine protease 27
1	4870	0.595	eS6_d1_28_E12		
1	8615	0.585	eS6_d1_29_A10		
1	8116	0.483	eS6_d1_30_H10	IABY01009517	
1	7465	0.537	eS6_d1_31_D10*		
1	8137	0.404	eS6_d1_32_D12	LOC107443710	cadherin-related tumor suppressor-like
1	8396	0.476	eS6_d1_32_G05*	LOC107452006	transcription factor AP-2-beta-like
1	1224	0.517	eS6_d1_33_C11	LOC107448603	uncharacterized LOC107448603
2	8704	0.530	eS6_d1_34_D05*	LOC107447504	homeobox protein Hox-B4a-like, ftz-B
2	4565	0.433	eS7_SB_035_D03	LOC107447504	homeobox protein Hox-B4a-like, ftz-B
2	11882	0.391	eS7_SB_035_D03	LOC107447504	homeobox protein Hox-B4a-like, ftz-B
2	6178	0.306	eS7_SB_037_E07	LOC107447504	homeobox protein Hox-B4a-like, ftz-B
2	6529	0.571	eS6_d1_35_F10	LOC107453461	argininosuccinate synthase-like
2	10205	0.554	eS6_d1_36_B07		
2	3126	0.592	eS6_d1_43_B11		
2	5110	0.509	eS6_d1_44_D10		
2	9590	0.588	eS6_d1_51_D07	LOC107456383	zinc finger protein 25
2	654	0.590	eS6_d1_51_H02	IABY01004033	
2	9647	0.587	eS6_d1_52_C02*	LOC107447988	homeobox protein engrailed-like ceh-16, Noto1
2	4037	0.597	eS6_d1_57_F09	LOC107456922	segment polarity protein dishevelled homolog DVL-3
2	7784	0.556	eS7_005_F03	LOC107456962	probable basic-leucine zipper transcription factor J
2	12252	0.591	eS7_SB_009_G07	IABY01005160	
2	2630	0.520	eS7_SB_011_D07*	LOC107456088	iroquois-class homeodomain protein IRX-6, mirr4
2	879	0.568	eS7_SB_011_D07*	LOC107456088	iroquois-class homeodomain protein IRX-6, mirr4
2	1216	0.581	S7_d1_29_C06	LOC107456088	iroquois-class homeodomain protein IRX-6, mirr4
2	8972	0.515	eS7_SB_018_F06	LOC107455065	zinc finger protein-like 1 homolog
2	4626	0.179	eS7_SB_028_C07*	LOC107445228	protein gooseberry, Prd2
2	5659	0.593	eS7_SB_030_B11		
2	8303	0.462	eS7_SB_035_C08	LOC107452623	small glutamine-rich tetratricopeptide repeat-containing protein beta-like
2	2111	0.501	eS7_SB_038_H11*	LOC107448645	transcription factor Sp9
2	7540	0.370	S7_d1_24_G01	LOC107448645	transcription factor Sp9
2	10234	0.465	eS7_SB_042_D01	LOC107454524	phospholipase A-2-activating protein
2	341	0.592	eS7_SB_043_C05		
2	6422	0.589	eS7_SB_047_C01*	LOC107437200	inosine-5′-monophosphate dehydrogenase 2
2	2691	0.440	S7_d1_04_F07		
2	2109	0.595	S7_d1_06_C11	LOC107457213	alpha-(1,3)-fucosyltransferase C
2	10975	0.504	S7_d1_08_C05	LOC107454396	bone morphogenetic protein receptor type-2
2	47	0.561	S7_d1_18_F06		
2	10933	0.545	S7_d1_19_D06		
2	3648	0.576	S7_d1_20_H05*	IABY01019901	
2	5931	0.583	S7_d1_21_H03	LOC107436591	NADH dehydrogenase [ubiquinone] flavoprotein 1, mitochondrial
2	1902	0.597	S7_d1_29_B03	LOC107437456	cytochrome P450 302a1, mitochondrial
2	6935	0.480	S7_d1_30_G04		
2	11999	0.528	S7_d1_33_A05	LOC107437124	lipoyltransferase 1, mitochondrial-like
2	9564	0.540	S7_d1_35_G02*	LOC107449043	toll-like receptor Tollo
2	9079	0.569	S7_d1_39_A09*		
2	1615	0.515	S7_d1_40_C07	LOC107441637	DNA replication licensing factor mcm4-A
2	2079	0.474	S7_d1_40_G11		

a Expression of the transcripts related to the EST clones indicated by asterisks (*) was examined by whole-mount *in situ* hybridization (see Fig. 1).

Supplementary video related to this article can be found at https://doi.org/10.1016/j.dib.2019.105088.

The following is the supplementary data related to this article:

**Movie S1. Time-lapse observation of *Pt-ptc* + *Pt-dpp* double pRNAi embryos.** These embryos were from the same egg sac that was used for RNA extraction in the microarray experiment. Time (day: h: min) after the start of recording (late stage 4) is indicated. The time point when *Pt-ptc* + *Pt-dpp* double pRNAi embryos were lysed for the RNA extraction was about 00:07:20. The time-lapse recording lasted more than two days, which should have covered the stages of germ band formation and elongation and limb bud formation. Apparently, the embryos observed failed to develop the orthogonal body axes and extra-embryonic tissues. The related phenotypes have been described in our previous work [1,2].

## 2 Experimental design, materials, and methods

### 2.1 Parental RNA interference (pRNAi)

The general procedure for pRNAi-mediated gene knockdown in *P. tepidariorum* was previously described [1]. Specifically, a mated female was injected with approximately 2.0 μl of *Pt-ptc* and *Pt-dpp* dsRNA mixture (0.6–1.0 μg/μl each) 5 times at the intervals of 2–3 days. The 709-bp (nt 1–709) region of *Pt-ptc* cDNA (GB_ACC: AB433900.1) and the 736-bp region (nt 1005–1740) of *Pt-dpp* cDNA (GB_ACC: AB096072.1) were used for the synthesis of the *Pt-ptc* and *Pt-dpp* dsRNAs, whose specific knockdown effects were previously described [1,2]. Embryos derived from an egg sac produced by the female two days before (normal) and 24 days after (*Pt-ptc* + *Pt-dpp* double pRNAi) the first injection of the dsRNA were used for RNA extraction. The morphological phenotype of the *Pt-ptc* + *Pt-dpp* double pRNAi embryos from the same egg sac that was used for the RNA extraction was recorded by time-lapse microscopy (Movie S1).

### 2.2 Microarray analysis

40-mer oligonucleotide probes designed were embedded in custom microarrays (CombiMatrix CustomArray 12K×2, CustomArray, Inc.). The same microarray design was used in our previous work [3]. The details of the custom microarray design including the probe sequences are available from the GEO database (GPL11390 and GPL11391). The total RNAs used for microarray analysis were extracted from approximately 250 embryos at late stage 5 using MagExtractor (Toyobo). The time point when *Pt-ptc* + *Pt-dpp* double pRNAi embryos were lysed for the RNA extraction was about 00:07:20 (day: h: min) in Movie S1. The RNA integrity was examined with an Agilent Bioanalyzer 2100. The cRNA labeled with Cy3 or Cy5 was prepared from 2 μg of total RNA using RNA Transcript SureLABEL Core Kit (Takara). The cRNA probes were hybridized to microarrays using Hybridization buffer (5× SSC, 0.1% SDS, 10% formamide) at 42 °C for 16–20 h. The microarray slides were scanned using a GenePix 4000B Scanner (Molecular Devices). There were no biological replicates. The obtained images were analyzed using an Array-Pro Analyzer ver. 4.5 (Media Cybernetics, Inc.). The quantitative data were subjected to Loess normalization. The ratio of the normalized intensity values ([*Pt-ptc* + *Pt-dpp* double pRNAi]/[normal]) for each array spot was calculated. The array spots for alpha-catenin (GB_ACC: AB433907; GI: LOC107439705), elongation factor 1-alpha (GB_ACC: AB433908; GI: LOC107441347), and histone H3 (GB_ACC: AB433909; GI, LOC107447866) served as negative controls (Table 1), while some of the array spots for *Pt-dpp* (GB_ACC: AB096072; GI: LOC107442925) and *Pt-cad* (GB_ACC: AB096075; GI: LOC107437910) were detected as positive, as expected from previous work [1,2]. The values from these positive and negative array spots validated the microarray experiment.

### 2.3 Embryo staining by whole-mount *in situ* hybridization (WISH)

Since most EST clones that were associated with positive array spots were instantly available, they were used for the synthesis of Digoxigenin-labeled RNA probes for WISH. The EST clone At_eW_022_P10 was used for the synthesis of fluorescein-labeled RNA probe, which marked the cumulus mesenchymal cells in stage 5 embryos [2]. Single- and double-staining of embryos at stages 5–8 by WISH were performed as described [1]. The stained embryos were photographed using a stereomicroscope (SZX12, Olympus) equipped with a color CCD camera (C7780-10, Hamamatsu Photonics).
